# Potatoes as Food

**Published:** 1878-01

**Authors:** 


### POTATOES AS FOOD.

It is undeniable that Americans eat too much meat, and we may as well have an eye to principle as well as price in the setting of our tables. It is not wise, as a general thing, to eat by rule, at the same time there is nothing blameworthy in eating scientifically, especially if it is clearly promotive of health and is at the same time much more economical, which is an important consideration with that large class of worthy people who live by their daily labor, the widow and the fatherless poor.

The most nutritious part of the potatoe is contained within the eighth of an inch immediately under the skin, so that in peeling, three-fourths of the most valuable portion is utterly wasted : the most healthful mode of preparation for the table is by baking; then all the water which does not unite with the starch is driven off, leaving it mealy and dry. If to be boiled, wash the potatoe clean; let it stand two or more hours in cold water, put it in a pot of water with some salt,boil quickly with the skin on, until the fork passes smoothly through the core; pour off all the water; set the pot over the fire, uncovered, for five minutes; remove the skin rapidly, and place on the table in a covered dish. When fried brown, in slices,the starch is turned into charcoal, indigestible and innutritious. If the potatoes are old, as in the Spring, they should be peeled and soaked in cold water, then thrown into boiling water, then served as before.

Sixty pounds of potatoes make a bushel and costs a dollar, but five pounds of meat at twenty cents a pound gives but one twelfth as much nutriment; or, to put it in another form, a pound of potatoes costing near two cents, warms and nourishes the human body as much as a pound of meat, which costs twenty cents; still, as meat is more easily digested than potatoes are and has some valuable ingredients peculiar to itself, the actual practical value of the two articles may be stated in terms thus: potatoes, as food, are one-third cheaper than meat, at the prices above stated.

Three-quarters of a pound of potatoes out of a whole pound is water; fresh, clear, lean meat is the same. The yield of different qualities of potatoes per acre, in the same soil and under the same cultivation will surprise many, as the following table will show, being the result of carefully conducted experiments by Dr. W. F. Hexamer of Westchester County, State of New York.

| | Bushels per acre. | | Bushels per acre. |
|---|---|---|---|
| Cuzco | 360 | White Mercer | 180 |
| Garnet Chili | 290 | Fluke | 160 |
| Pink-eye Rusty Coat | 280 | Prince Albert | 160 |
| Peach Blow | 240 | Early June | 150 |
| White Peach Blow | 230 | White Rock | 130 |
| Prairie Seedling | 230 | Early Dykeman | 120 |
| Blue Mercer | 220 | Early Cottage | 110 |
| "Buckley's Seedling" | 210 | Early Sovereign | 80 |
| Buckeye | 200 | Rough and Ready | 56 |

The whole farming world would be increased debtors to Dr. Hexamer's scientific industry if he will institute another set of experiments to ascertain how much nutrition the principle kinds above named contain; such information would be of special practical worth to both producer and consumer, for if the last variety in the table has no more nutriment than the first and will keep as well, the difference in profit to the farmer would be very great. While it does not seem to pay for the trouble of putting the cut side of the potatoe downwards in planting, there is a difference of yield of nearly one-fifth in favor of large seed.

